# Integrated Multi-Omics Analysis for Inferring Molecular Players in Inclusion Body Myositis

**DOI:** 10.3390/antiox12081639

**Published:** 2023-08-19

**Authors:** Judith Cantó-Santos, Laura Valls-Roca, Ester Tobías, Clara Oliva, Francesc Josep García-García, Mariona Guitart-Mampel, Félix Andújar-Sánchez, Anna Esteve-Codina, Beatriz Martín-Mur, Joan Padrosa, Raquel Aránega, Pedro J. Moreno-Lozano, José César Milisenda, Rafael Artuch, Josep M. Grau-Junyent, Glòria Garrabou

**Affiliations:** 1 Inherited Metabolic Disorders and Muscular Diseases Research Group, Institut d’Investigacions Biomèdiques August Pi i Sunyer (IDIBAPS) and Faculty of Medicine and Health Sciences, University of Barcelona, 08036 Barcelona, Spain; jcanto@recerca.clinic.cat (J.C.-S.); lvalls@recerca.clinic.cat (L.V.-R.); etobiasb@clinic.cat (E.T.); fjgarcia@recerca.clinic.cat (F.J.G.-G.); mguitart@recerca.clinic.cat (M.G.-M.); fandujsa7@alumnes.ub.edu (F.A.-S.); padrosa@clinic.cat (J.P.); aranega@clinic.cat (R.A.); pjmoreno@clinic.cat (P.J.M.-L.); 2 Department of Internal Medicine, Hospital Clinic of Barcelona, 08036 Barcelona, Spain; 3 CIBERER—Spanish Biomedical Research Centre in Rare Diseases, 28029 Madrid, Spain; 4 Department of Clinical Biochemistry, Institut de Recerca Sant Joan de Déu, Esplugues de Llobregat, 08950 Barcelona, Spain; clara.olivam@sjd.es (C.O.); rartuch@sjdhospitalbarcelona.org (R.A.); 5 CNAG-CRG, Centre for Genomic Regulation, Barcelona Institute of Science and Technology, 08028 Barcelona, Spain; anna.esteve@cnag.eu (A.E.-C.); beatriz.martin@cnag.eu (B.M.-M.); 6 Department of Medicine and Health Sciences, Universitat Pompeu Fabra (UPF), 08003 Barcelona, Spain

**Keywords:** inclusion body myositis (IBM), metabolism, organic acids, nucleotides, biomarker

## Abstract

Inclusion body myositis (IBM) is an acquired inflammatory myopathy affecting proximal and distal muscles that leads to weakness in patients over 50. It is diagnosed based on clinical and histological findings in muscle related to inflammation, degeneration, and mitochondria. In relation to IBM, a shortage of validated disease models and a lack of biomarkers and effective treatments constitute an unmet medical need. To overcome these hurdles, we performed an omics analysis of multiple samples from IBM patients (saliva, fibroblasts, urine, plasma, and muscle) to gain insight into the pathophysiology of IBM. Degeneration was evident due to the presence of amyloid β peptide 1–42 (Aβ1–42) in the saliva of the analyzed IBM patients. The presence of metabolic disarrangements in IBM was indicated by an imbalanced organic acid profile in fibroblasts and urine. Specifically, abnormal levels of L-pyroglutamic and orotic acid were supported by the abnormal expression of related metabolites in plasma and urine (glutathione and pyrimidines) and the aberrant expression of upstream gene regulators (L2HGDH, IDH2, OPLAH, and ASL) in muscle. Combined levels of L-pyroglutamic and orotic acid displayed an outstanding biomarker signature in urine with 100% sensitivity and specificity. The confirmation of systemic metabolic disarrangements in IBM and the identification of novel biomarkers reported herein unveil novel insights that require validation in larger cohorts.

## 1. Introduction

IBM is an acquired inflammatory myopathy (IM) affecting patients over 50 years old. Its prevalence ranges from 50–180 per million [1,2]. The first symptoms arise in the quadriceps and finger flexors, manifesting as complications in climbing stairs and grasping objects. These symptoms evolve into proximal and distal muscle weakness that eventually impairs ambulation within 10–15 years from the disease’s onset. Moreover, 60% of patients are also affected by dysphagia [3,4]. Diagnosis is based on clinical and histological findings at the inflammatory, degenerative, and mitochondrial levels. Inflammation is triggered by the infiltration of CD8+ cytotoxic T-cells in myofibers and the upregulation of major histocompatibility complex I (MHC-I). Degeneration is presented in the form of rimmed vacuoles and the inclusion of TDP43, p62, and amyloid protein deposits. In mitochondria, ragged-red fibers, complex IV (COX-negative) fibers from the mitochondrial respiratory chain (MRC), and complex II (SDH positive) fibers are displayed, which are also present in primary mitochondrial diseases and mitochondrial myopathies [1,5,6,7].

IBM-related inflammation is critical in the disease and is the reason it is grouped within IMs such as dermatomyositis, polymyositis, anti-synthetase syndrome, necrotizing myopathy, and non-specific myositis. Indeed, apoptosis-resistant highly differentiated and cytotoxic CD8+ T cells (with the KLRG1 receptor) might trigger the onset of IBM [1,3,8], yet it is unclear whether the trigger is inflammatory or degenerative. However, inflammatory treatments like immunosuppressants and immunomodulatory treatments that are effective for other IMs are ineffective for IBM. Ongoing clinical trials pertaining to IBM involve, for example, the ability of a monoclonal antibody against the KLRG1 receptor to deplete highly differentiated and cytotoxic CD8+ T cells (clinical trial code: NCT04659031), a rapamycin (previously approved to prevent rejection in organ transplantation and to treat certain types of cancer) that blocks effector T cells while preserving regulatory T cells and inducing autophagy (NCT04789070), and even stem cell muscle injections (NCT04975841) [1].

Considering mitochondrial damage in IBM, it has been speculated that it could be the result of pro-inflammatory cytokine release (IL-1β, TNF-α, etc.) that could increase oxidative, nitrosative, and endoplasmic-reticulum-related stress. Such lesions may endanger mitochondrial membrane permeability and transport, leading to a dysfunctional MRC that would accumulate mtDNA mutations [1,3,9]. Dysfunctional mitochondria may be aggravated by insufficient autophagy, specifically mitophagy, further increasing inflammation via mitochondrial damage. All these factors contribute to a positive feedback loop that may eventually trigger muscle fiber atrophy and accelerated muscle aging [1,6]. To avoid or reduce the lesions produced via increased oxidative stress, several antioxidant defense systems are activated, including superoxide dismutases (SOD) and glutathione (GSH) redox systems [10]. An increase in the levels of GSH, which is catalyzed by GSH peroxidase (GPX) enzymes, is usually associated with the higher production of oxidants and electrophiles in cells [11,12]. Consequently, the lack of sufficient GSH antioxidant activity may aggravate the outcome of the disease [10,11,12]. Thus, some GSH analogs have been tested as therapeutics, while decreasing GSH activity has been proven effective as an anti-tumor target, making tumors more susceptible to chemotherapy or radiotherapy [11]. None of these antioxidant therapies, neither metabolic strategies,, have ever been tested in relation to IBM, even though IBM displays pathological traits of mitochondrial function and some metabolic disorders often exhibit muscle involvement.

Targeting treatment efficacy can be enhanced through the incorporation of validated IBM disease models. So far, most of the pathophysiology of IBM has been revealed through the analysis of biological samples from the target tissue (muscle histopathology studies, RNA-seq, etc.) or peripheral tissues (mainly lymphocyte RNA-seq and metabolome phenotyping) [5,7,13,14]. A mouse xenograft containing IBM muscle biopsies [15] served as a breakthrough in the field, facilitating the in vivo evaluation of disease pathophysiology. The major contribution of this model was the finding that rimmed vacuoles resisted after the murine xenografts were treated with anti-CD3+, which reduced CD8+ T cell levels and impaired MHC-I upregulation, thus supporting the idea that new treatments should target degeneration to treat muscle weakness. Another relevant model of IBM was developed and validated by our group [13] using patient-derived fibroblasts. This model recapitulated the main hallmarks of the target tissue of the disease (inflammatory, degenerative, and metabolic muscle imbalances). Impaired mitochondrial function in the IBM fibroblasts was similar to that of primary mitochondrial diseases [7,16] and was present in a peripheral tissue outside of muscle, thereby confirming molecular disturbances at the systemic level. A relevant finding of the study was an abnormal organic acid profile in fibroblasts associated with MRC dysfunction and oxidative stress. This alteration uncovered the potential use of metabolites and fibroblasts to identify biomarkers needed to monitor disease progression. However, neither this nor other studies have screened these markers in the non-invasive fluids of IBM patients.

The same study highlighted the usefulness of omics technology with respect to seeking novel molecular players in the disease that may emerge as surrogate biomarkers or therapeutic targets. Indeed, the identification of genetic variants, proteins, or metabolites associated with the onset and progression of several diseases has been achieved on account of the development of unbiased, high-throughput omics technologies [17]. These analyses provide massive amounts of data based on a small quantity of biological material and a reduced number of measurements. In addition, omics can be combined (e.g., RNA-seq, target metabolomics, WES, etc.) and integrated to obtain a broad view of a disease’s pathophysiology [17].

Thus, in the present article, we aimed to identify novel target molecules and pathways in IBM that may prove to be non-invasive biomarkers, focusing on the mitochondrial and metabolic profile of the disease, by performing an omics analysis (targeted metabolome and RNA seq analyses) of different biological samples isolated from IBM patients (saliva, urine, plasma, fibroblasts, and muscle).

## 2. Materials and Methods

### 2.1. Study Design

A case–control study was conducted from 2018 to 2022 in the Department of Internal Medicine at the Hospital Clínic of Barcelona (Barcelona, Spain). Patients were diagnosed as suffering from IBM according to clinical and pathological tests performed at our hospital after fulfilling the criteria proposed by the European Neuromuscular Centre for IBM diagnosis [18]. Exclusion criteria were age < 40 years old; family history of hereditary mitochondrial disease; comorbidities and concomitant infections; or drug abuse. IBM patients participating in the study signed informed consent forms previously approved by the Ethical Committee of our hospital (code HCB/2017/0808). CTLs were recruited from among healthy volunteers—who were age-and gender-paired with IBM patients—after excluding any comorbidities or pathological processes, and they signed the corresponding informed consent forms.

### 2.2. Sample Collection

Saliva samples were passively collected (no stimulation) in sterile plastic containers, to which 0.5 mg of sodium azide (Sigma #S2002, Saint Louis, MO, USA) and 0.5 mg of thioflavin S (Sigma #T1892, Saint Louis, MO, USA) were added to prevent bacterial growth and Aβ42 aggregation, respectively [19,20,21]. Nine IBM patients and 5 CTLs were enrolled. Samples were immediately frozen at −80 °C until use.

Fibroblasts were obtained using a skin punch biopsy from 14 cases and 12 controls. Cells were grown in 25 mM glucose DMEM supplemented with 10% FBS and 1% penicillin–streptomycin at 37 °C in a humidified 5% CO_2_ air incubator (all from Gibco, Waltham, MA, USA). Cells were harvested and collected using trypsin (Gibco, Waltham, MA, USA) at 80% confluence and characterized at passages 3 to 10.

Urine was collected from 6 cases and 6 controls, and organic acid and nucleotide content was analyzed and normalized with reference to creatinine levels.

Plasma was collected from 12 patients and 12 controls and used to determine GPX activity.

Muscle biopsies were performed for diagnostic purposes [22,23]. Leftover biopsy material from 5 IBM patients and 6 CTLs (defined as individuals without histological myopathic alterations) were included in this study to perform an mRNA seq analysis.

### 2.3. Aβ1–42 Detection

To detect Aβ1–42 in saliva, 50 µL per sample was added to an enzyme-linked immunosorbent assay (ELISA) plate per duplicate (Biomatik #EKU02331, Kitchener, ON, Canada) according to the manufacturer’s protocol.

### 2.4. Metabolite Quantification

Total protein amounts in fibroblast samples were quantified using a bicinchoninic acid protein assay (BCA) (Thermo Scientific #23255, Waltham, MA, USA), and 5 mg/mL was resuspended in 200 µL of PBS and centrifuged (1500× *g*; 10 min) to collect the supernatant. Urine samples were normalized according to their creatinine content. Organic acids were extracted with ethyl acetate and diethyl ether and derived with bis(trimethylsilyl) trifluoro-acetamide, as previously reported [24]. The trimethylsilyl derivatives obtained were separated using gas chromatography (Agilent 7890A, Wilmington, DE, USA) and detected in a mass spectrometer (Agilent 5975C, Wilmington, DE, USA). The results were expressed as *m/z* ratios.

### 2.5. Glutathione Peroxidase (GPX) Activity Assay

To quantify the GPX activity, 10 µL of plasma per sample was used in an enzymatic assay (MAK437, Sigma-Aldrich, Saint Louis, MO, USA), as specified in the manufacturer’s protocol.

### 2.6. RNA Extraction and mRNA Library Preparation and Sequencing

Total RNA was isolated from the muscle via tissue homogenization (OMNI Tissue, Dallas, TX, USA) with TriPure (Roche #11667165001, Basel, Switzerland) and isopropanol (Sigma #190764, Saint Louis, MO, USA) precipitation protocols. Total content was assessed using a Quawell UV-VIS Spectrophotometer Q5000. Concentrations were adjusted to 100 ng/µL in RNase-free water. The quality control of the total RNA was conducted via the Qubit RNA HS Assay (Thermo Scientific #Q32852, Waltham, MA, USA) and the RNA 6000 Nano Assay using a Bioanalyzer 2100 (Agilent, Santa Clara, CA, USA). The RNA-seq libraries were prepared using the TruSeq Stranded mRNA LT Sample Prep Kit. Briefly, total RNA (500 ng) was enriched in terms of its mRNA fraction and fragmented. Strand-specificity was achieved by ensuring that the second-strand cDNA incorporated dUTPs instead of dTTPs. The blunt-ended double-stranded cDNA was 3’-adenylated, and Illumina-platform-compatible adaptors with unique dual indexes and unique molecular identifiers (Integrated DNA Technologies, Coralville, IA, USA) were ligated. The ligation product was enriched with 15 PCR cycles, and the final library was validated on a 2100 Bioanalyzer (Agilent #G2939BA, Santa Clara, CA, USA) using the DNA 7500 assay. The libraries were sequenced on HiSeq 4000 System (Illumina, San Diego, CA, USA) with a read length of 2 × 51 bp using the HiSeq 4000 SBS kit (Illumina #FC-410-1002, San Diego, CA, USA). Primary data analysis, image analysis, base calling, and quality scoring of the run were processed using the manufacturer’s software, namely, Real Time Analysis, (RTA 2.7.7) followed by the generation of FASTQ sequence files.

### 2.7. RNA-Seq Data Processing and Analysis

RNA-seq reads were mapped against the human reference genome (GRCh38) using STAR software version 2.5.3a [25] with ENCODE parameters. Annotated genes were quantified using human GENCODE annotation file version 34 with RSEM v1.3.0 [26] and default parameters. Differential expression analysis was performed using the DESeq2 V1.26.0 R package [27], using a Wald test to compare affected and control groups and adjusting for sex in the model. Genes were considered differentially expressed with an adjusted *p*-value of < 0.05 and an absolute fold change of |FC|> 1.5. Differentially expressed genes (DEGs) were filtered according to the genes found in in the Metabolism pathway of the Mitocarta 3.0 [28]. On the Metaboanalyst platform, two analyses were performed, namely, a joint pathway analysis and network analysis, both combining transcriptomics and targeted metabolomics lists with their fold values [29].

### 2.8. Statistical Analysis

Statistical analysis was performed using the Statistical Package for the Social Sciences software version 27 (IBM SPSS Statistics; IBM Corp, Armonk, NY, USA) and GraphPad Prism 9 (GraphPad Software, San Diego. CA, USA). Results were expressed as means ± SEM or as FC between conditions, normalized according to creatinine or protein load. Obtained data were compared between independent experiments using the non-parametric Mann–Whitney U test or Kruskal–Wallis’s test. Receiver Operating Characteristic (ROC) curves and the area under the ROC curve (AUC) were examined to determine biomarker performance. The AUC ranges from 0 to 1, where values > 0.7 reflect good biomarker performance. Sensitivity and specificity values were determined via a binary logistic regression analysis using the Wald test method. The signification threshold was set at *p*-value of < 0.05.

## 3. Results

### 3.1. Clinical and Epidemiological Data

In this study, the cohort of IBM patients was composed of white Caucasians ranging from 40 to 83 years old, while the controls (CTLs; healthy individuals at the time of inclusion) were recruited from the same population and ranged from 38 to 79 years old. Both cohorts were paired by age and sex. The number of samples per cohort, the male vs. female ratio, and the mean age in each cohort can be found in Table 1.

### 3.2. Degeneration Markers: Aβ1–42 Concentration Was Higher in IBM Samples

**Figure 1:**
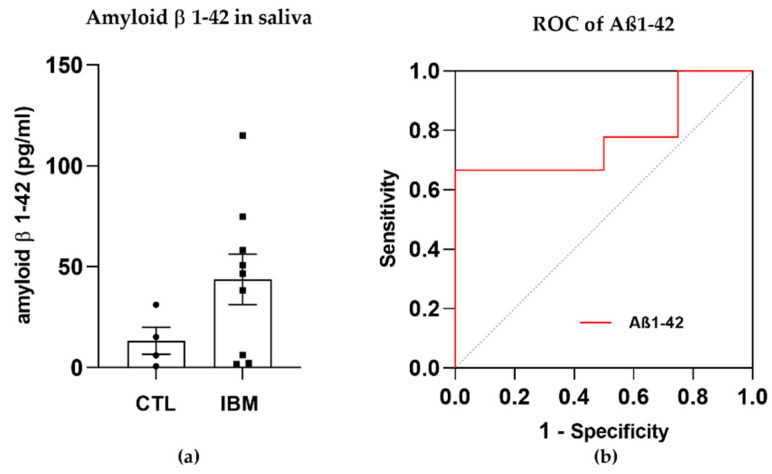
Amyloid β peptide 1–42 (Aβ1–42) in saliva samples. (**a**) Bar graphs of the concentration of Aβ1–42 in inclusion body myositis (IBM) vs. control (CTL) samples. (**b**) Receiver operating characteristic (ROC) curve and area under the ROC curve (AUC) of Aβ1–42 (AUC = 0.78 ± 0.13. *p*-value = 0.12), yielding sensitivity and specificity scores of 66.70% and 50.00%, respectively. Higher Aβ1–42 concentrations and high AUC scores in the IBM group suggested that this peptide could aid in discriminating between cohorts.

The presence of amyloid β in rimmed vacuoles and protein depots of muscle tissue from IBM patients has been recently investigated [30]. Although this long-standing belief is currently in a state of controversy, we previously confirmed that there were altered levels of amyloidogenic degenerative markers in plasma isolated from IBM patients (including BACE-1 and PS-1 [31]) and decided to evaluate the presence of Aβ1–42 (associated with β- and ɣ-secretase enzymes) in a fluid that was easily accessible, i.e., saliva, for which samples isolated from IBM patients vs. CTLs were compared. Interestingly, Aβ1–42 displayed trends towards higher concentrations in the IBM cohort (43.7 ± 12.5 vs. 13.3 ± 6.7, *p*-value = 0.15) (Figure 1a) and significant (i.e., 66.70% and 50.00%) sensitivity and specificity scores as a surrogate marker of disease, with a high AUC of 0.78 ± 0.13 (*p*-value = 0.12) (Figure 1b and Table S1). Although these results should be validated in larger cohorts, Aβ1–42 has emerged as a promising marker to potentially discriminate between cohorts.

### 3.3. Mitochondrial Alterations

#### 3.3.1. Organic Acid Profiles Increased in IBM Fibroblasts

**Figure 2:**
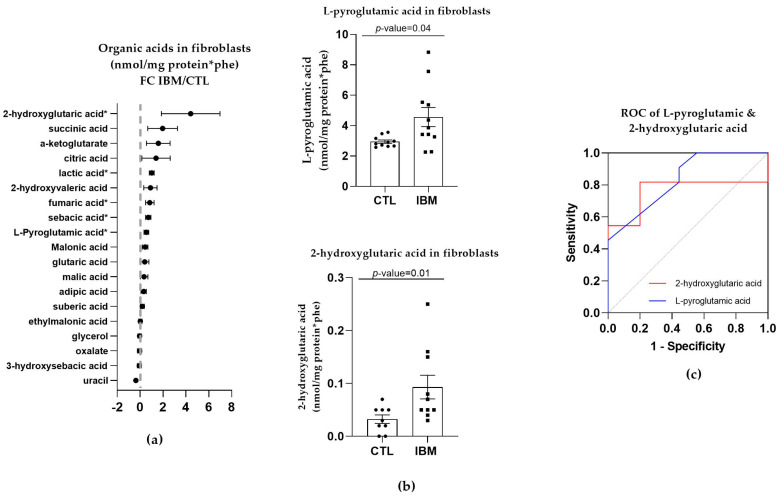
Organic acid profiles in inclusion body myositis (IBM) vs. control (CTL) fibroblasts (*n* = 11 vs. 10). (**a**) Fold change (FC) of the concentration of organic acids in IBM vs. CTL fibroblasts (* *p*-value < 0.05). (**b**) Bar graph of L-pyroglutamic and 2-hydroxyvaleric acids (*p*-value < 0.05). (**c**) Receiver operating characteristic (ROC) curve and area under the ROC curve (AUC) of L-pyroglutamic (AUC = 0.79 ± 0.12. *p*-value = 0.03) and 2-hydroxyvaleric acids (AUC = 0.84 ± 0.09. *p*-value = 0.01). Their sensitivity and specificity values are 72.7% and 80% for L-pyroglutamic acid and 72.70% and 77.80% for 2-hydroxy glutaric acid. All the organic acids presented increased concentrations in the IBM vs. CTL fibroblasts, among which L-pyroglutamic and 2-hydroxy glutaric acids were the most remarkable, with the former being associated with glutathione synthesis and the latter with the TCA cycle.

Targeted metabolome characterization of the organic acids in fibroblasts represented by the FC between the IBM and CTL fibroblasts revealed that there were higher concentrations of organic acids in the IBM group (as shown in Figure 2a). Such an aberrant profile suggests altered intermediary metabolism potentially related to MRC dysfunction. Among the organic acids identified, we focused on L-pyroglutamic and 2-hydroxy glutaric acids (4.6 ± 0.6 vs. 3.0 ± 0.1, *p*-value = 0.04 and 0.2 ± 0.1 vs. 0.03 ± 0.01, *p*-value = 0.01, respectively) (Figure 2b), which are associated with GSH synthesis and the tricarboxylic acid cycle (TCA) cycle, respectively. These organic acids may also be detected in non-invasive fluids such as urine. Their ROC curves were significant in both cases. L-pyroglutamic acid showed an AUC = 0.76 ± 0.12 (*p*-value = 0.04) accompanied by significant sensitivity (72.7%) and specificity (80%) values, and 2-hydroxy glutaric acid showed a high AUC = 0.84 ± 0.09 for (*p*-value = 0.01), with 72.70% and 77.80% sensitivity and specificity values (Figure 2c and Table S1). Thus, the next step was to evaluate the signatures of organic acids in other peripheral samples of the IBM patients, which could be used as a liquid biopsy for the evaluation of disease. To promote non-invasive sample profiling, we decided to examine the organic acids in urine.

#### 3.3.2. Organic Acid Profiling in Urine Reveals Altered Metabolism in IBM

**Figure 3:**
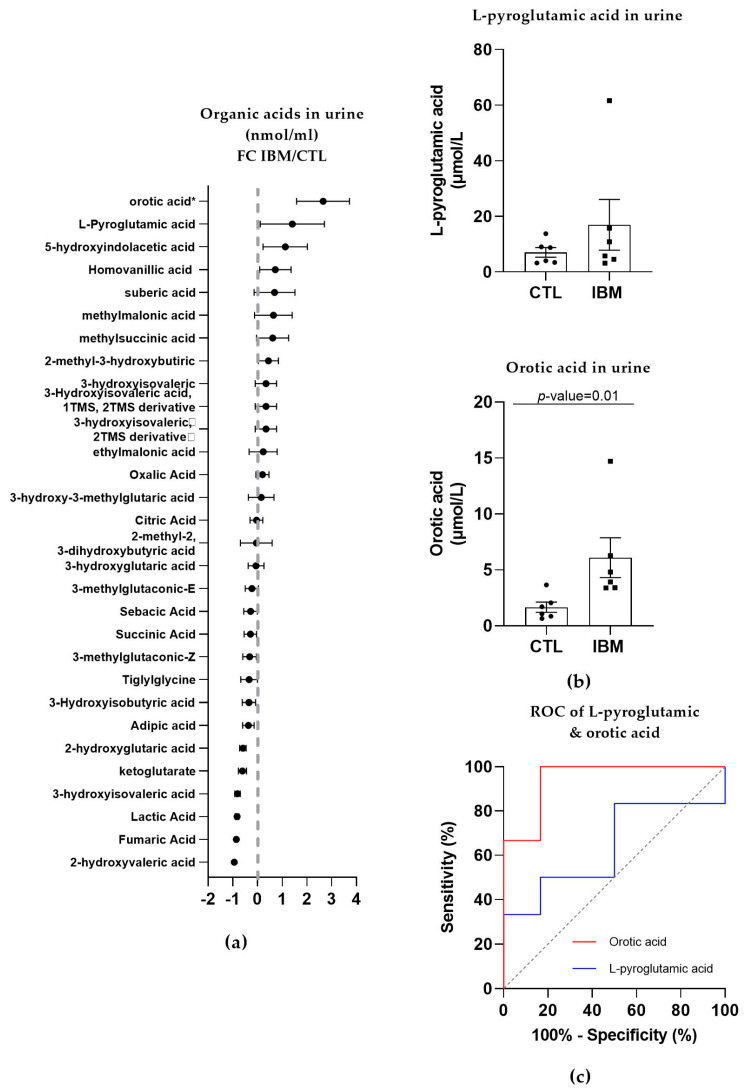
Organic acid profiles in the urine of inclusion body myositis (IBM) vs. control (CTL) samples (*n* = 6/group). (**a**) Fold change (FC) of the concentration of organic acids in the urine of lBM vs. CTLs. (**b**) Bar graphs of L-pyroglutamic and orotic acids (*p*-value < 0.05). (**c**) Receiver operating characteristic (ROC) curve and area under the ROC curve (AUC) of L-pyroglutamic (AUC = 0.64 ± 0.17. *p*-value = 0.42) and orotic acids (AUC = 0.94 ± 0.07. *p*-value = 0.01). Their sensitivity and specificity values are 50.0% and 83.3% for L-pyroglutamic acid and 100.0% and 83.3% for orotic acid, but these values changed to 100% sensitivity and specificity when both acids were tested together. The presence of altered organic acids in urine corroborated the imbalanced organic acid profile in fibroblasts and highlights L-pyroglutamic and orotic acid as potential fluid biomarkers.

In the urine of the IBM patients, roughly half of the organic acids were upregulated, while the other half presented a reduction, as shown in Figure 3a. The most relevant organic acids were L-pyroglutamic acid (as in fibroblasts; 17.0 ± 9.1 vs. 7.0 ± 1.7, *p*-value = 0.48) and orotic acid (6.1 ± 1.8 vs. 1.7 ± 0.5, *p*-value = 0.01, Figure 3b), which are associated with GSH synthesis and the pyrimidine biosynthetic pathway, respectively. Their ROC curves presented an AUC = 0.64 ± 0.17 (*p*-value = 0.42, with 50% sensitivity and 83.3% specificity) for L-pyroglutamic acid and AUC = 0.94 ± 0.07 (*p*-value = 0.01) for orotic acid, with 100% sensitivity and 83.3% specificity scores (Figure 3c and Table S1). These sensitivity and specificity values increased to 100% when L-pyroglutamic acid and orotic acid were tested together. The presence of organic acids alterations in urine and fibroblasts confirmed an imbalanced organic acid profile, thus identifying L-pyroglutamic and orotic acid as potential fluid biomarkers in this disease.

#### 3.3.3. Nucleotide Synthesis Precursor Levels Were Increased in the Urine of IBM Patients

**Figure 4:**
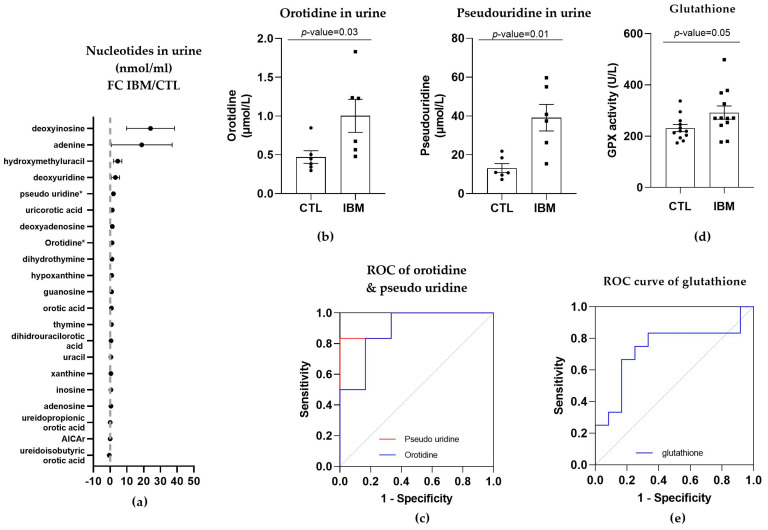
Nucleotides in urine and glutathione in plasma of inclusion body myositis (IBM) vs. control (CTL) samples (*n* = 6/group). (**a**) Fold change (FC) of the concentration of nucleotides in urine of IBM patients vs. CTLs. (**b**) Bar graphs of orotidine and pseudo-uridine (*p*-value < 0.05). (**c**) Receiver operating characteristic (ROC) curve and area under the ROC curve (AUC) of orotidine (AUC = 0.89 ± 0.10. *p*-value = 0.02) and pseudo-uridine (AUC = 0.94 ± 0.07. *p*-value = 0.01). Their sensitivity and specificity values are 66.7% and 83.3% for orotidine and 83.3% for both in pseudo-uridine. (**d**) Glutathione peroxidase (GPX) activity in IBM vs. CTL plasma samples (*p*-value = 0.05). (**e**) Receiver Operating Characteristic (ROC) curve and area under the ROC curve (AUC) of GPX (AUC = 0.74 ± 0.11, *p*-value = 0.05), with 66.7% sensitivity and 75.0% specificity. Almost all nucleotides were present in higher concentrations in the urine isolated from the IBM patients, with orotidine and pseudo-uridine being statistically significant, thereby corroborating the altered orotic acid biosynthesis previously observed. GPX activity was also higher in the IBM patients, suggesting increased glutathione redox system activity associated with oxidative stress.

As orotic acid plays a role in the pyrimidine biosynthetic pathway, we evaluated nucleotide metabolism in urine in the IBM vs. CTL samples. Briefly, almost all pyrimidines and purines were present in higher concentrations in the urine isolated from the IBM patients (Figure 4a), with orotidine and pseudo-uridine being statistically significant (1.0 ± 0.2 vs. 0.5 ± 0.1, *p*-value = 0.03, and 39.1 ± 6.8 vs. 13.2 ± 2.3, *p*-value = 0.01, respectively). These two metabolites are associated with the pyrimidine biosynthesis pathway, with orotidine being the product of orotic acid plus 5-phosphoribosyl-1-pyrophosphate (PRPP) catalyzed by uridine monophosphate synthetase (UMPS) in its orotate phosphoribosyl transferase activity, and pseudo-uridine being the product of orotidine catalyzed by UMPS in its orotidine-5′-phosphate decarboxylase (OMP decarboxylase) activity [32].

The quantification of these two pyrimidines (Figure 4b) and the evaluation of their ROC curves displayed an AUC= 0.89 ± 0.10 (*p*-value = 0.02) for orotidine, with 66.7% sensitivity and 83.3% specificity, and an AUC = 0.94 ± 0.07 (*p*-value = 0.01) for pseudo-uridine, with 83.3% sensitivity and specificity (Figure 4c and Table S1), thus corroborating the altered orotic acid biosynthesis previously observed.

#### 3.3.4. GPX Activity Levels in Plasma

The increase in L-pyroglutamic acid in the fibroblasts and urine could be associated with GSH metabolism. Thus, we examined the activity of GPX to investigate the antioxidant defense system activity in the plasma of the IBM vs. CTL samples.

GPX activity was increased in the IBM samples (292.0 ± 25.9 vs. 231.4 ± 13.7, *p*-value = 0.05, Figure 4d), presenting significant sensitivity and specificity scores (66.7% and 75.0%, respectively), with a high AUC of 0.74 ± 0.11 (*p*-value = 0.05) (Figure 4e and Table S1). This assay revealed that there was a higher level of activity in the IBM samples, which was potentially required to compensate for an increase in oxidative stress, which has previously been reported to contribute to neuromuscular disease physiopathology [33].

#### 3.3.5. Metabolic-Upstream-Related Genes in the IBM Muscle Transcriptome

**Figure 5:**
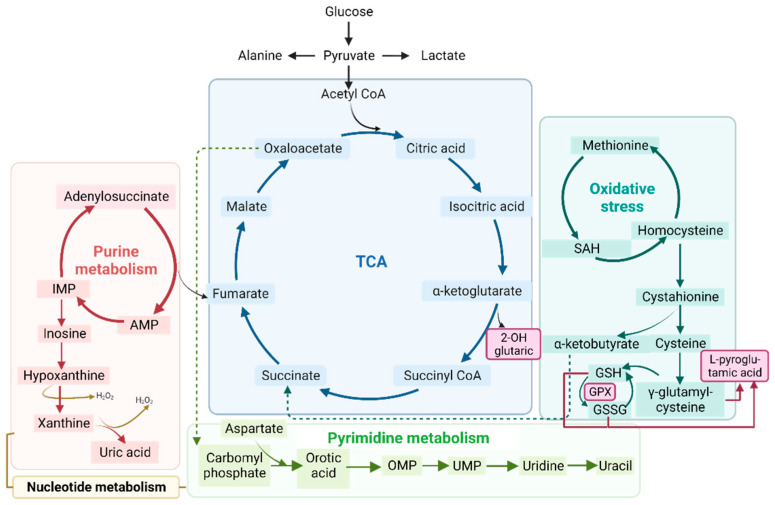
Schematic representation of the tricarboxylic acid cycle (TCA) (in blue), purine (in red) and pyrimidine (in green) metabolism (both part of nucleotide metabolism), and oxidative stress (in turquoise). All of these processes are related to the metabolic profile examined in the organic acid, nucleotide, and RNA seq analyses. Abbreviations: IMP: inosine monophosphate; AMP: adenosine monophosphate; OMP: orotate monophosphate; UMP: uridine 5’-monophosphate; SAH: S-adenosylhomocysteine; GSH: glutathione; GSSG: glutathione disulfide; GPX: glutathione peroxidase.

All metabolic disarrangements led to the identification of target metabolites related to nucleotides, TCA, and the glutathione system in different samples from the IBM patients (Figure 5); as these metabolites presented high values of specificity and sensibility (Table S1). Thus, we wanted to link the gene expression in the target tissue to the detected metabolites in fibroblasts and urine to determine whether there were systemic metabolic changes. Therefore, we filtered the DEGs related to metabolism from the IBM muscle transcriptome, and found multiple metabolic DEGs.. Specifically, of the 418 DEGs in the IBM patients related to mitochondria [28], 197 were associated with metabolism [28], 32 of which were upregulated, while 165 were downregulated (Table S2). The top upregulated DEGs were OXCT2, KMO, MTHFD1L, CYP27B1, and AIFM3, in descending order, whereas the most downregulated DEGs were ACOT11, ALDH6A1, BCO2, GPT2, and SLC25A30, in ascending order. In detail, OXCT2 is involved in ketone body catabolism; KMO is involved in tryptophan metabolism; MTHFD1L is involved in purine biosynthesis; CYP27B1 is involved in calcium homeostasis; and AIFM3 is involved in apoptosis, while ACOT11 plays a role in fatty acid and CoA metabolism; ALDH6A1 plays a role in valine and pyrimidine catabolic pathways; BCO2 plays a role in in vitamin A biosynthesis; GPT2 plays a role in in pyruvate metabolism; and SLC25A30 plays a role in in mitochondrial membrane transport. These data support the notion of functional metabolic deregulation at the genetic level and confirm the relevance of metabolic deregulation in relation to this disease.

**Figure 6:**
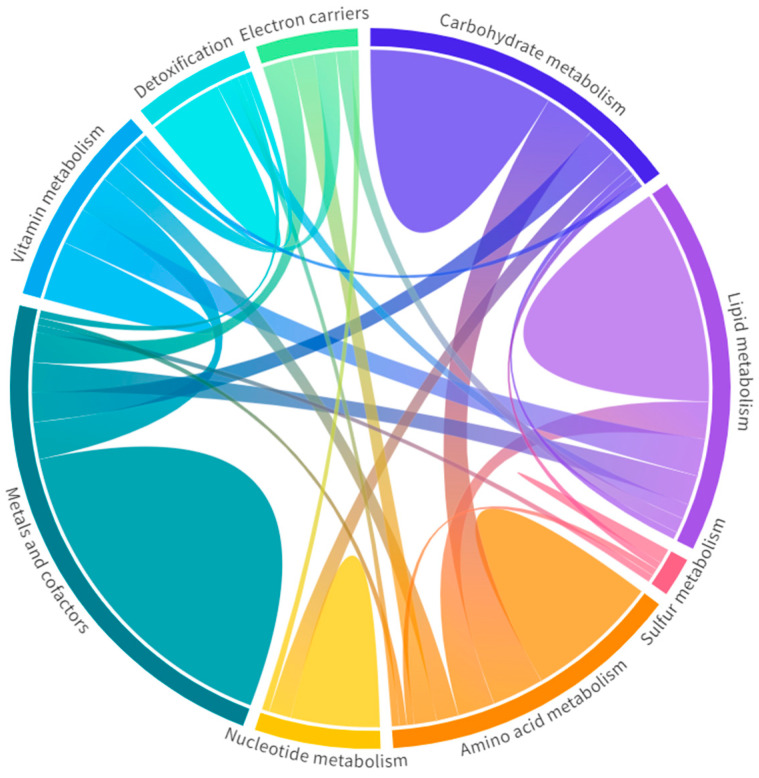
Metabolic pathways in the muscle RNA-seq of inclusion body myositis (IBM) patients vs. controls (CTLs). Each pathway is represented with a different color with the genes unique to that pathway (domes) and the interactions of genes across pathways (chords). Wider domes and chords represent a higher number of genes. Among them, carbohydrate metabolism and metals and cofactors, with 56.8% and 52.8% of DEGs involved, were the most affected metabolic pathways (Table S2). Overall, these data support the notion of functional metabolic deregulation at the gene expression level and confirm the relevance of metabolic deregulation in relation to this disease.

Specifically, these 197 metabolism-related genes were clustered in nine Mito Pathways [28], which are carbohydrate, lipid, amino acid, nucleotide, vitamin, and sulfur metabolism; detoxification; electron carriers; and metals and cofactors, as represented in Figure 6. Among them, the top affected Mito Pathways were carbohydrate metabolism, with 56.8% of DEGs involved (42/74 DEGs in common), and metals and cofactors, with 52.8% (65/123 DEGs matched) [28] (Table S3). In all pathways, more DEGs were downregulated than upregulated. In addition, some genes were involved in more than one pathway, as shown in Figure 6, revealing how the metabolism is interconnected and every alteration could be directly or indirectly associated with a specific dysfunction.

### 3.4. Gene–Metabolites Interaction: Potential Interactome Framework for IBM Samples

**Figure 7:**
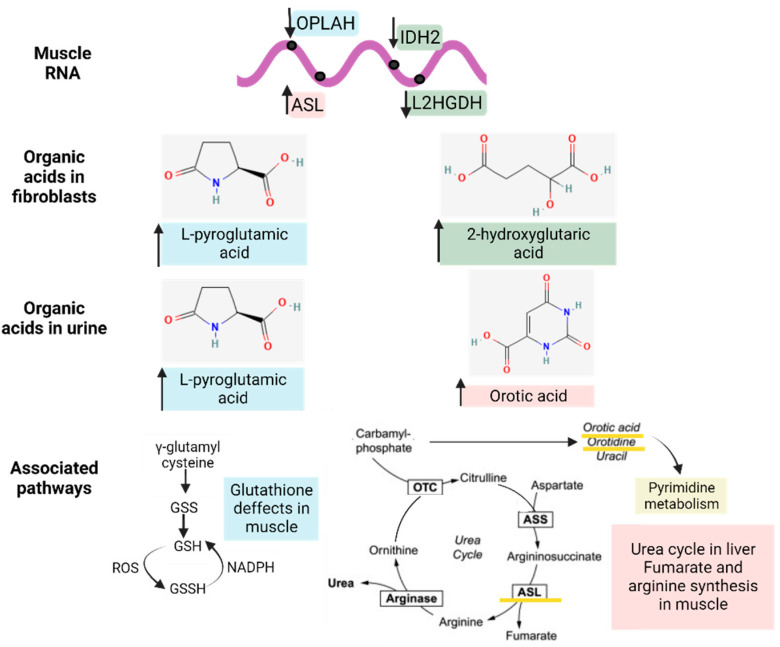
Interactome of the metabolic differentially expressed genes (DEGs) in muscle related to the significantly altered organic acid patterns (in fibroblasts and urine) and their associated pathways. The arrows next to the genes and metabolites refer to their increased or decreased expression in IBM samples. The different colors (blue, green, and pink) indicate the upstream (genes) and downstream (organic acids and nucleotides) effectors of each pathway.

To reveal whether metabolic DEGs may be related to deregulated organic acid profiles in urine and, potentially, biomarkers of disease, we linked the previously relevant organic acids in fibroblasts and urine to their respective DEGs at the muscle level, as represented in Figure 7. In brief, 2-hydroxy glutaric acid, whose levels were increased in fibroblasts, was associated with L-2 and D-2-hydroxy glutaric aciduria genes (L2HGDH and IDH2), which were downregulated in muscle tissue, as expected, and are found in primary 2-Hydroxy glutaric aciduria disease (ORPHA:19) [34]. In the case of L-pyroglutamic acid, whose levels were increased in fibroblasts and urine, it was associated with the 5-Oxoprolinase ATP-Hydrolyzing (OPLAH) gene, which, accordingly, was downregulated in muscle tissue, as is the case in primary 5-Oxoprolinuria disease (ORPHA:289846) [34,35]. Downregulated OPLAH is related to GSH defects in muscle tissue, which leads to an increased concentration of L-pyroglutamic acid in urine. Regarding orotic acid, whose levels were increased in urine, it was associated with the Argininosuccinate Lyase (ASL) gene, which was upregulated in muscle tissue, as expected, as is the case in primary Argininosuccinic aciduria disorder (ORPHA:23) [34]. ASL is part of the urea cycle but also plays a role in fumarate and arginine synthesis in muscle. All these multi-omics and interactome connections between metabolites and genes confirm the impact of metabolic dysregulation in IBM.

In parallel to the associations presented in Figure 7, we also performed a Metaboanalyst analysis [29] combining DEGs related to metabolism in muscle vs. organic acids and nucleotides in urine. This approach allows for the identification of interactions by connecting gene expression to metabolite data. Concisely, when compared against DEGs in metabolism, 10 pathways in organic acids and 7 in nucleotides were statistically significant (FDR < 0.05). All seven pathways enriched in nucleotide analysis were also enriched in organic acids, with the TCA cycle being the most relevant pathway (represented in Figure 5), followed by valine, leucine, and isoleucine degradation; propanoate metabolism; and pyruvate metabolism (Table S4). In the network enrichment analysis, the TCA cycle was the most enriched network, followed by alanine, aspartate, and glutamate metabolism (FDR < 0.05) in metabolic DEGs combined with organic acids, while purine metabolism and pyrimidine metabolism were the significantly enriched networks (FDR < 0.05) in the metabolic DEGs combined with nucleotides (Table S4). In short, the data reinforced the interconnection of all affected metabolic pathways and the relevance of metabolic changes in this disease.

## 4. Discussion

Few biomarkers associated with diagnosis or prognosis in IBM have been found despite the development of omics technologies and the high-throughput screening of several samples and patients [17]. In IBM, the major advances in biomarker identification include the description of the HLA haplotypes HLA-DRB1*03:01, HLA-DRB1*01:01, and HLA-DRB1*13:01 [1,36], with the last two being IBM-specific alleles; the discovery of IBM-specific CD8+ KLRG1+ T cells [8,37]; the identification of the NT5c1A autoantibody in the sera of IBM patients [38,39]; the detection of the overexpression of cadherin 1 (CDH1) in muscle tissue from IBM patients [40]; and the finding of the type I and γ interferon pathways (detected at the transcriptomic and proteomic levels) [41], which supported the immune interaction between immune cells and myofibers in IBM. Of note, none of the previous biomarker signatures are related to the degenerative or mitochondrial changes in IBM, nor are they real-time measures that can be evaluated in liquid biopsies to reflect disease evolution or prognosis.

This study aimed to perform multi-omics profiling through the high-throughput analysis of numerous biological samples from IBM patients to infer novel target molecules with a differential profile in patients with this disease. We first explored Aβ1–42 measurements in IBM patient saliva that yielded relevant but non-significant conclusions that should be more comprehensively explored in studies with larger cohorts. Interestingly, our group’s previous findings depicting aberrant levels of BACE1 and PSN1 proteins in the plasma of IBM patients may confirm the systemic impact of the disease and the implication of amyloidogenesis in IBM [31].

Subsequently, the fact that IBM mitochondrial alterations resemble mitochondrial primary and secondary diseases [5,7,13,16] led to our analysis of the metabolic profiles in the IBM samples (first in fibroblasts and then in urine). Briefly, the targeted metabolic profile of the IBM fibroblasts displayed a higher expression of organic acids in the IBM samples, revealing L-pyroglutamic and 2-hydroxy glutaric acid (the latter of which is closely associated with the TCA cycle) as the most remarkable acids. When the organic acid profile was examined in the subjects’ urine and more metabolites were detected, L-pyroglutamic acid and orotic acid emerged as a surrogate biomarker of this disease. Orotic acid can be increased in different metabolic conditions involving urea cycle disorders and pyrimidine biosynthesis defects. Carriers of these conditions may display subtle orotic acid increments in their urine, but considering that these are rare diseases, it is unlikely that our cohort of IBM patients harbors variants in genes relates to these metabolic pathways. Pyroglutamic acid excretion in urine can increase due to genetic but also environmental conditions [35,42], such as special diets, drug treatments, prematurity, or malnutrition. Although we did not rule out the presence of these factors in our cohort of patients, the increased pyroglutamic acid values observed in fibroblasts as well, which is not influenced by environmental conditions, strongly support the feasibility of this surrogate biomarker for the study of IBM patients. To understand these organic acid alterations in this disease, we determined the GSH content and nucleotide levels in urine due to their association with L-pyroglutamic acid and orotic acid metabolism, respectively. GPX activity was increased in the IBM plasma samples, suggesting a need to counterbalance oxidative stress in cases of IBM [13,33]. Specifically, GPX catalyzes the following reaction, namely, GSH + H_2_O_2_ → GSSG + H_2_O (where GSSG is GSH disulfide), to maintain redox cell homeostasis by attracting free radicals to reduced GSH and to convert them into GSSG and H_2_O. Related to orotic acid, the levels of orotidine and pseudo-uridine, from the pyrimidine biosynthetic pathway, were increased in the IBM patients’ urine samples. These findings reinforce the metabolic and oxidant component of IBM, which is probably also present in other IMs [33].

When the metabolic profile was associated with the metabolically related DEGs in the muscle RNA-seq analysis, we observed a correlation between some DEGs and organic acids, thus confirming the value of L-pyroglutamic acid and orotic acid as potential biomarker signatures in IBM urine and the relevance of metabolism to this disease. Specifically, the interactome analysis revealed that the following Mito pathways are affected in this disease: carbohydrate, lipid, amino acid, nucleotide, vitamin, and sulfur metabolism; detoxification; electron carriers; and metals and cofactors. In addition to Mito pathways, the Metaboanalyst analysis combining metabolic DEGs in muscle with organic acids and nucleotides in urine revealed that the TCA cycle; valine, leucine, and isoleucine degradation; propanoate metabolism; and pyruvate metabolism were the most enriched pathways, thereby corroborating the mitochondrial and metabolic role in muscle DEGs.

Previously, other studies also found increased nucleotide synthesis precursors and altered pyrimidine and GSH metabolism through IBM pathway analysis in blood metabolomes [5]. We associated these alterationswith L-pyroglutamic acid and orotic acid. Indeed, the IBM pathway analysis of the blood metabolomes also resembles the Metaboanalyst pathway and network analysis that we observed in relation to IBM in the urine and muscle samples, thus supporting the RNA-seq–metabolome link in the muscle vs. fluid samples (urine and blood) and the robustness of the present findings. However, previous analyses were only conducted at the transcriptomic level; this is the first study analyzing metabolites in the non-invasive fluids of IBM patients.

Most of the biomarkers identified displayed high sensitivity and specificity scores and prompted the concept of potential support in diagnosis and follow-up of patients via a non-invasive approach. Among them, we propose that L-pyroglutamic combined with orotic acid serves as a surrogate biomarker of disease in urine samples due to their high sensitivity (100%) and specificity (100%) scores when tested together. Independently, L-pyroglutamic acid is a biomarker in Eosinophilic Esophagitis (in urine) and Glutathione Synthetase Deficiency (in urine and blood). Chronically high levels of L-pyroglutamic acid are associated with several inborn errors of metabolism, like 5-Oxoprolinuria, 5-oxoprolinase deficiency, Glutathione Synthetase Deficiency, Hakinsinuria, and Propionic acidemia [43]. Similarly, orotic acid in urine is a biomarker of the following metabolic diseases: Ornithine Transcarbamylase Deficiency, N-Acetylglutamate Synthase Deficiency, and Orotic Aciduria [44]. None of these have been associated with muscular diseases, thus avoiding potential overlap with clinical symptoms between these conditions. These results should be confirmed in a larger cohort, and future studies should validate their potential use as a prognostic marker in accordance with disease evolution.

Despite the novelty of these results and their translational relevance for clinical intervention, we should acknowledge some limitations of our study. The main limitation is the small sample size of the study, which was mainly governed by the fact that IBM is a rare disease for which it is difficult to access biological samples. Interestingly, the intertwined and narrow relationship between the results obtained across the different analyses and tissues, made these findings stronger and validated the usefulness of multi-omics approaches in identifying biomarkers in disease.

Specifically, combining metabolite with RNA-seq profiles (Figure 7) revealed that L-pyroglutamic acid, whose levels were increased in fibroblasts and urine, was associated with downregulated OPLAH gene, which is related to GSH defects in muscle and is responsible for an increased concentration of L-pyroglutamic acid in urine [35]. Orotic acid, whose levels were increased in urine, was associated with the ASL gene, part of the urea cycle, which is only metabolically completed in the liver but plays a role in fumarate and arginine synthesis in muscle. These associations should be confirmed in studies with larger cohorts, but the results display the emergent value of the reported metabolites along with their high sensitivity and specificity scores, their potential to support the diagnosis and follow-up of patient management, the systemic impact of this disease (previously thought to be restricted to muscle), and the relevance of metabolism in IBM (which was thought to be only minor and secondary). It is still unclear whether these findings could guide future treatment strategies, but the implication of metabolism and oxidative stress in IBM is now undeniable.

In future studies, the metabolic profiling of the same patients at different time points of the disease could contribute to a better understanding of the disease’s evolution and the evaluation of reported biomarkers for disease prognosis, especially considering that IBM is a progressive disease and yet some patients experience little or no disease progression through periods of 4 to 12 years [3]. A better screening of IBM evolution could also aid in the stratification of patients according to their progression rate and in consideration of the hypothesis that IBM is a spectrum disorder [38]. Similarly, the use of these biomarkers in response to treatment efficacy could also yield interesting conclusions.

In conclusion, multi-omics profiling is a powerful tool for revealing disease-specific metabolic dysregulation. By performing metabolic and RNA-seq profiling of fibroblasts, urine, plasma, and muscle samples from IBM patients, we have identified potential biomarker signatures combining organic acids and gene expression associations. This preliminary study revealed promising metabolic fingerprints for examining disease progression and treatment efficacy.

## 5. Conclusions

- Metabolic dysregulation in IBM is present outside the target tissue (muscle), as seen in the altered organic acids in fibroblasts and urine;
- The multi-omics profiling of patients’ samples allows for the evaluation of disease-associated phenotypes, constituting an untargeted approach enabling the potential detection of novel molecular players;
- The detection of L-pyroglutamic and orotic acids in urine displayed an outstanding biomarker signature, with 100% sensitivity and specificity;
- The validation of potential biomarkers in non-invasive samples like urine may eventually aid in the screening of patients’ disease progression and treatment efficacy.

## Figures and Tables

**Table 1:** Clinical and epidemiological data concerning the cohorts involved in the study.

Sample Type	Summary	Num. of Samples (*n*)	Male/Female Ratio	Mean Age (Years)
Fibroblasts	IBM	14	0.6	66.9 ± 3.6
	CTL	12	0.4	57.4 ± 4.6
Urine	IBM	6	0.5	70.8 ± 3.7
	CTL	6	0.5	71.2 ± 3.4
Muscle	IBM	5	0.40	62.6 ± 6.4
	CTL	6	0.67	44.6 ± 4.7
Saliva	IBM	9	0.44	67.2 ± 4.7
	CTL	5	0.6	57.0 ± 5.9
Plasma	IBM	12	0.6	63.7 ± 3.5
	CTL	12	0.6	75.3 ± 0.9

Data presented as means ± SEM. Abbreviations: IBM: Inclusion Body Myositis patients. CTL: healthy individuals. There was an age- and gender-paired distribution between cohorts.

## Data Availability

The data presented in this study are available in the article, supplementary material or on request from the corresponding authors.

## Supplementary Materials

The following supporting information can be downloaded at: https://www.mdpi.com/article/10.3390/antiox12081639/s1, Table S1: Biomarker performance values for target molecules; Table S2: Differentially expressed genes (DEGs) involved in metabolism (extracted from Mitocarta 3.0) determined from RNA-seq in the muscle of IBM patients (*n* = 5) vs. CTLs (*n* = 6), Table S3: Metabolic Mito Pathways along with the number of differentially expressed genes (DEGs) involved (from IBM muscle RNA-seq); Table S4: Metaboanalyst analysis combining metabolic differentially expressed genes (DEGs) in muscle RNA-seq with organic acids and nucleotides in urine. Significant pathways from pathway analysis and network enrichment analysis are represented in the table.
