# Supplementary material for: Integrated Multi-Omics Analysis for Inferring Molecular Players in Inclusion Body Myositis
Source: Antioxidants (Basel). 2023 Aug 19;12(8):1639. doi: 10.3390/antiox12081639 (PMC10452026; doi:10.3390/antiox12081639)
Supplement: Supplementary file 1 [file antioxidants-12-01639-s001.zip › antioxidants-2515072-supplementary.pdf]

Table S1. Biomarker performance values for target molecules

Target molecule	AUC ± SD	p-value	Sensitivity	Specificity	Index
Aβ1-42	0.8 ± 0.1	0.12	66.7%	50.0%	61.5%
Orotic acid	0.9 ± 0.1	0.01	100.0%	83.3%	91.7%
2-Hydroxyglutaric acid	0.8 ± 0.1	0.01	72.7%	77.8%	75.0%
L-pyroglutamic acid (fibroblasts)	0.8 ± 0.1	0.04	72.7%	80.0%	76.2%
L-pyroglutamic acid (urine)	0.6 ± 0.2	0.42	50.0%	83.3%	66.7%
Orotidine	0.9 ± 0.1	0.02	66.7%	83.3%	75.0%
Pseudouridina	0.9 ± 0.1	0.01	83.3%	83.3%	83.3%
Glutathione	0.7 ± 0.1	0.05	66.7%	75.0%	70.8%
L-pyroglutamic (urine) + orotic acid			100%	100%	100%

Abbreviations: AUC: area under the Receiver Operating Characteristic (ROC) curve; SD: standard deviation; Index: mean of the sensitivity and specificity.

Table S2. Differentially expressed genes (DEGs) involved in metabolism (extracted from Mitocarta 3.0) from RNA seq in muscle of inclusion boy myositis (IBM) patients (n=5) vs controls (CTL) (n=6).

**Gene ID**	**Gene name**	**log2 Fold change**	**p-value adj (FDR)**
GPAM	Glycerol-3-Phosphate Acyltransferase. Mitochondrial	-1.45	6.91E-10
GOT2	Glutamic-Oxaloacetic Transaminase 2	-1.17	4.66E-09
NNT	Nicotinamide Nucleotide Transhydrogenase	-1.48	4.84E-08
ACADM	Acyl-CoA Dehydrogenase Medium Chain	-1.34	1.38E-07
MTHFD1L	Methylenetetrahydrofolate Dehydrogenase (NADP+ Dependent) 1 Like	3.13	2.44E-06
LIPT2	Lipoyl(Octanoyl) Transferase 2	-1.16	3.82E-06
SUCLA2	Succinate-CoA Ligase ADP-Forming Subunit Beta	-1.04	3.87E-06
NDUFS1	NADH:Ubiquinone Oxidoreductase Core Subunit S1	-1.16	4.30E-06
COQ7	Coenzyme Q7. Hydroxylase	-0.99	4.59E-06
FXN	Frataxin	-1.00	5.17E-06
ACOT11	Acyl-CoA Thioesterase 11	-2.90	5.23E-06
ALDH6A1	Aldehyde Dehydrogenase 6 Family Member A1	-1.82	5.37E-06
PDP2	Pyruvate Dehydrogenase Phosphatase Catalytic Subunit 2	-0.88	8.51E-06
BCKDHB	Branched Chain Keto Acid Dehydrogenase E1 Subunit Beta	-0.86	9.69E-06
PRXL2A	Peroxiredoxin Like 2A	-0.70	1.10E-05
PRDX3	Peroxiredoxin 3	-0.70	1.24E-05
DHTKD1	Dehydrogenase E1 And Transketolase Domain Containing 1	-1.21	1.38E-05
PDHX	Pyruvate Dehydrogenase Complex Component X	-0.92	1.61E-05
SLC25A23	Solute Carrier Family 25 Member 23	-1.05	1.78E-05
DLD	Dihydrolipoamide Dehydrogenase	-0.77	1.96E-05
FDXR	Ferredoxin Reductase	1.34	2.03E-05
SFXN3	Sideroflexin 3	1.47	2.32E-05
ACADSB	Acyl-CoA Dehydrogenase Short/Branched Chain	-1.22	2.50E-05
SDHC	Succinate Dehydrogenase Complex Subunit C	-0.77	3.71E-05
RDH13	Retinol Dehydrogenase 13	-1.31	4.83E-05
SUCLG2	Succinate-CoA Ligase GDP-Forming Subunit Beta	-0.87	5.04E-05
COQ10A	Coenzyme Q10A	-0.95	5.44E-05
OXCT2	3-Oxoacid CoA-Transferase 2	5.46	6.10E-05
MCCC2	Methylcrotonyl-CoA Carboxylase Subunit 2	-1.05	6.66E-05
SLC25A30	Solute Carrier Family 25 Member 30	-1.64	6.92E-05
MT-CO1	Mitochondrially Encoded Cytochrome C Oxidase I	-1.12	1.12E-04
COQ8A	Coenzyme Q8A	-1.15	1.19E-04
MTARC2	Mitochondrial Amidoxime Reducing Component 2	-0.78	1.25E-04
L2HGDH	L-2-Hydroxyglutarate Dehydrogenase	-1.23	1.29E-04
MAOB	Monoamine Oxidase B	-1.29	1.31E-04
HADH	Hydroxyacyl-CoA Dehydrogenase	-1.01	1.33E-04
SLC25A4	Solute Carrier Family 25 Member 4	-0.87	1.54E-04
CS	Citrate Synthase	-0.90	1.77E-04
PCK2	Phosphoenolpyruvate Carboxykinase 2. Mitochondrial	1.75	1.78E-04
IBA57	Iron-Sulfur Cluster Assembly Factor IBA57	-0.93	1.97E-04
DBT	Dihydrolipoamide Branched Chain Transacylase E2	-0.97	2.21E-04
HCCS	Holocytochrome C Synthase	-0.83	2.68E-04
ALDH5A1	Aldehyde Dehydrogenase 5 Family Member A1	-1.18	2.95E-04
ETFDH	Electron Transfer Flavoprotein Dehydrogenase	-1.07	3.49E-04
MT-CYB	Mitochondrially Encoded Cytochrome B	-1.03	3.51E-04
COX10	Cytochrome C Oxidase Assembly Factor Heme A:Farnesyltransferase COX10	-0.95	3.74E-04
SLC25A38	Solute Carrier Family 25 Member 38	-0.78	4.01E-04

CHDH	Choline Dehydrogenase	-1.41	4.07E-04
CKMT2	Creatine Kinase. Mitochondrial 2	-0.93	4.39E-04
ACO2	Aconitase 2	-0.97	4.47E-04
ACSL6	Acyl-CoA Synthetase Long Chain Family Member 6	-1.39	4.62E-04
PDPR	Pyruvate Dehydrogenase Phosphatase Regulatory Subunit	-1.20	4.63E-04
COQ6	Coenzyme Q6. Monooxygenase	-0.81	4.81E-04
PCBD2	Pterin-4 Alpha-Carbinolamine Dehydratase 2	-0.90	4.89E-04
FH	Fumarate Hydratase	-0.58	5.16E-04
PCCB	Propionyl-CoA Carboxylase Subunit Beta	-0.72	5.17E-04
SIRT5	Sirtuin 5	-1.08	5.49E-04
COQ5	Coenzyme Q5. Methyltransferase	-0.84	5.60E-04
CPT1C	Carnitine Palmitoyltransferase 1C	1.25	5.92E-04
MMUT	Methylmalonyl-CoA Mutase	-0.83	6.31E-04
LIAS	Lipoic Acid Synthetase	-0.64	7.87E-04
ACAT1	Acetyl-CoA Acetyltransferase 1	-1.03	8.41E-04
ACACA	Acetyl-CoA Carboxylase Alpha	-0.85	9.76E-04
CYP11A1	Cytochrome P450 Family 11 Subfamily A Member 1	0.91	1.15E-03
CBR3	Carbonyl Reductase 3	1.42	1.21E-03
CPOX	Coproporphyrinogen Oxidase	-0.63	1.35E-03
COQ9	Coenzyme Q9	-0.70	1.37E-03
SDHD	Succinate Dehydrogenase Complex Subunit D	-0.61	1.40E-03
DLAT	Dihydrolipoamide S-Acetyltransferase	-1.04	1.40E-03
TSTD1	Thiosulfate Sulfurtransferase Like Domain Containing 1	1.61	1.49E-03
BDH1	3-Hydroxybutyrate Dehydrogenase 1	-1.54	1.51E-03
GPT2	Glutamic--Pyruvic Transaminase 2	-1.73	1.60E-03
OGDH	Oxoglutarate Dehydrogenase	-0.76	1.71E-03
GRPEL1	GrpE Like 1. Mitochondrial	-0.60	1.80E-03
SLC25A12	Solute Carrier Family 25 Member 12	-1.08	1.97E-03
OXR1	Oxidation Resistance 1	-1.18	1.99E-03
HTD2	Hydroxyacyl-Thioester Dehydratase Type 2	-1.27	2.16E-03
PPOX	Protoporphyrinogen Oxidase	0.48	2.16E-03
PRELID1	PRELI Domain Containing 1	1.31	2.17E-03
NUBPL	NUBP Iron-Sulfur Cluster Assembly Factor. Mitochondrial	-0.73	2.18E-03
CROT	Carnitine O-Octanoyltransferase	-0.58	2.30E-03
FECH	Ferrochelatase	-0.71	2.36E-03
ETFA	Electron Transfer Flavoprotein Subunit Alpha	-0.59	2.36E-03
PNPLA8	Patatin Like Phospholipase Domain Containing 8	-0.56	2.42E-03
AIFM3	Apoptosis Inducing Factor Mitochondria Associated 3	2.44	2.46E-03
HADHB	Hydroxyacyl-CoA Dehydrogenase Trifunctional Multienzyme Complex Subunit Beta	-1.08	2.47E-03
ACSL1	Acyl-CoA Synthetase Long Chain Family Member 1	-1.18	2.48E-03
DMGDH	Dimethylglycine Dehydrogenase	-1.52	2.54E-03
SIRT4	Sirtuin 4	-0.96	2.58E-03
PNPO	Pyridoxamine 5'-Phosphate Oxidase	-0.61	2.61E-03
ACSS3	Acyl-CoA Synthetase Short Chain Family Member 3	-1.09	2.61E-03
SUOX	Sulfite Oxidase	-0.78	2.62E-03
MSRB3	Methionine Sulfoxide Reductase B3	-1.20	2.67E-03
NMNAT3	Nicotinamide Nucleotide Adenylyltransferase 3	-0.71	2.89E-03
SCP2	Sterol Carrier Protein 2	-0.70	3.01E-03
PLPBP	Pyridoxal Phosphate Binding Protein	-0.48	3.18E-03
NAT8L	N-Acetyltransferase 8 Like	-1.03	3.23E-03
SDHB	Succinate Dehydrogenase Complex Iron Sulfur Subunit B	-0.65	3.40E-03
TXNRD1	Thioredoxin Reductase 1	-0.56	3.43E-03
HSPA9	Heat Shock Protein Family A (Hsp70) Member 9	-0.79	3.58E-03
PHYH	Phytanoyl-CoA 2-Hydroxylase	-0.76	3.61E-03

IDI1	Isopentenyl-Diphosphate Delta Isomerase 1	-0.81	3.96E-03
SLC25A51	Solute Carrier Family 25 Member 51	-0.57	4.55E-03
AK3	Adenylate Kinase 3	-1.01	4.92E-03
SERAC1	Serine Active Site Containing 1	-0.61	5.27E-03
ACOT7	Acyl-CoA Thioesterase 7	1.44	5.29E-03
MTO1	Mitochondrial TRNA Translation Optimization 1	-0.41	5.65E-03
SLC25A5	Solute Carrier Family 25 Member 5	0.95	5.88E-03
QDPR	Quinoid Dihydropteridine Reductase	-0.64	5.91E-03
NUDT19	Nudix Hydrolase 19	-0.88	6.06E-03
DGUOK	Deoxyguanosine Kinase	0.59	6.41E-03
HOGA1	4-Hydroxy-2-Oxoglutarate Aldolase 1	1.76	6.50E-03
HIBADH	3-Hydroxyisobutyrate Dehydrogenase	-0.64	6.58E-03
ACACB	Acetyl-CoA Carboxylase Beta	-1.12	6.68E-03
PRELID3A	PRELI Domain Containing 3A	1.37	6.80E-03
MDH2	Malate Dehydrogenase 2	-0.70	7.10E-03
HSD17B4	Hydroxysteroid 17-Beta Dehydrogenase 4	-0.56	7.21E-03
NDUFS7	NADH:Ubiquinone Oxidoreductase Core Subunit S7	-0.69	7.35E-03
ISCA1	Iron-Sulfur Cluster Assembly 1	-0.58	7.43E-03
CISD1	CDGSH Iron Sulfur Domain 1	-0.63	7.99E-03
ADHFE1	Alcohol Dehydrogenase Iron Containing 1	-1.12	8.04E-03
IDH2	Isocitrate Dehydrogenase (NADP(+)) 2	-0.70	8.04E-03
LYRM4	LYR Motif Containing 4	0.65	8.11E-03
AGPAT4	1-Acylglycerol-3-Phosphate O-Acyltransferase 4	1.14	8.30E-03
KMO	Kynurenine 3-Monooxygenase	3.81	8.42E-03
DHRS1	Dehydrogenase/Reductase 1	0.83	8.78E-03
ALDH4A1	Aldehyde Dehydrogenase 4 Family Member A1	-0.83	8.99E-03
NME6	NME/NM23 Nucleoside Diphosphate Kinase 6	-0.51	9.21E-03
CYP27B1	Cytochrome P450 Family 27 Subfamily B Member 1	2.92	9.21E-03
NUDT8	Nudix Hydrolase 8	-0.67	9.22E-03
AKR1B10	Aldo-Keto Reductase Family 1 Member B10	1.42	9.32E-03
BCO2	Beta-Carotene Oxygenase 2	-1.75	9.55E-03
MTHFD2L	Methylenetetrahydrofolate Dehydrogenase (NADP+ Dependent) 2 Like	-0.72	1.02E-02
SLC25A37	Solute Carrier Family 25 Member 37	-0.76	1.05E-02
CYB5R3	Cytochrome B5 Reductase 3	0.58	1.09E-02
OSBPL1A	Oxysterol Binding Protein Like 1A	-0.81	1.14E-02
FDX1	Ferredoxin 1	-0.54	1.25E-02
AUH	AU RNA Binding Methylglutaconyl-CoA Hydratase	-0.64	1.26E-02
ALAS1	5'-Aminolevulinate Synthase 1	-0.66	1.27E-02
CRAT	Carnitine O-Acetyltransferase	-0.78	1.36E-02
PDHB	Pyruvate Dehydrogenase E1 Subunit Beta	-0.46	1.43E-02
EPHX2	Epoxide Hydrolase 2	-0.57	1.43E-02
SDHA	Succinate Dehydrogenase Complex Flavoprotein Subunit A	-0.68	1.43E-02
SLC25A3	Solute Carrier Family 25 Member 3	-0.48	1.48E-02
GLYCTK	Glycerate Kinase	1.06	1.50E-02
PRDX4	Peroxiredoxin 4	0.80	1.52E-02
SCO2	Synthesis Of Cytochrome C Oxidase 2	1.39	1.54E-02
GUK1	Guanylate Kinase 1	0.65	1.55E-02
SUGCT	Succinyl-CoA:Glutarate-CoA Transferase	-0.83	1.57E-02
GCSH	Glycine Cleavage System Protein H	-0.54	1.65E-02
HIBCH	3-Hydroxyisobutyryl-CoA Hydrolase	-0.41	1.66E-02
IDH3A	Isocitrate Dehydrogenase (NAD(+)) 3 Catalytic Subunit Alpha	-0.61	1.67E-02
PDK2	Pyruvate Dehydrogenase Kinase 2	-0.88	1.74E-02
ACAD8	Acyl-CoA Dehydrogenase Family Member 8	-0.53	1.85E-02
CYCS	Cytochrome C. Somatic	-0.70	1.90E-02

MGST3	Microsomal Glutathione S-Transferase 3	-0.69	1.92E-02
MPC2	Mitochondrial Pyruvate Carrier 2	-0.69	1.94E-02
NME4	NME/NM23 Nucleoside Diphosphate Kinase 4	-0.63	1.94E-02
NEU4	Neuraminidase 4	2.21	2.04E-02
NDUFS8	NADH:Ubiquinone Oxidoreductase Core Subunit S8	-0.62	2.07E-02
COX11	Cytochrome C Oxidase Copper Chaperone COX11	-0.67	2.20E-02
ABCB7	ATP Binding Cassette Subfamily B Member 7	-0.51	2.23E-02
CBR4	Carbonyl Reductase 4	-0.56	2.29E-02
ACADVL	Acyl-CoA Dehydrogenase Very Long Chain	-0.88	2.40E-02
BCKDHA	Branched Chain Keto Acid Dehydrogenase E1 Subunit Alpha	-0.74	2.42E-02
CHCHD7	Coiled-Coil-Helix-Coiled-Coil-Helix Domain Containing 7	-0.44	2.43E-02
SUCLG1	Succinate-CoA Ligase GDP/ADP-Forming Subunit Alpha	-0.51	2.45E-02
PDSS2	Decaprenyl Diphosphate Synthase Subunit 2	-0.41	2.47E-02
PDK1	Pyruvate Dehydrogenase Kinase 1	-0.65	2.48E-02
DHODH	Dihydroorotate Dehydrogenase (Quinone)	-0.61	2.72E-02
ZADH2	Prostaglandin Reductase 3	-0.47	2.81E-02
NADK2	NAD Kinase 2. Mitochondrial	-0.74	2.81E-02
CYC1	Cytochrome C1	-0.55	2.86E-02
IDH3B	Isocitrate Dehydrogenase (NAD(+)) 3 Non-Catalytic Subunit Beta	-0.44	3.05E-02
CA5B	Carbonic Anhydrase 5B	0.90	3.05E-02
FAHD1	Fumarylacetoacetate Hydrolase Domain Containing 1	-0.54	3.09E-02
SLC25A11	Solute Carrier Family 25 Member 11	-0.51	3.18E-02
ABHD10	Abhydrolase Domain Containing 10. Depalmitoylase	-0.44	3.31E-02
TMLHE	Trimethyllysine Hydroxylase. Epsilon	-0.71	3.51E-02
DLST	Dihydrolipoamide S-Succinyltransferase	-0.63	3.51E-02
NDUFV2	NADH:Ubiquinone Oxidoreductase Core Subunit V2	-0.44	3.58E-02
MLYCD	Malonyl-CoA Decarboxylase	-1.03	3.69E-02
CPT1B	Carnitine Palmitoyltransferase 1B	-1.02	3.71E-02
SIRT3	Sirtuin 3	-0.58	3.81E-02
IVD	Isovaleryl-CoA Dehydrogenase	-0.57	3.90E-02
UQCRFS1	Ubiquinol-Cytochrome C Reductase. Rieske Iron-Sulfur Polypeptide 1	-0.42	3.93E-02
ALDH2	Aldehyde Dehydrogenase 2 Family Member	-0.80	4.00E-02
MGST1	Microsomal Glutathione S-Transferase 1	1.18	4.07E-02
NDUFC2	NADH:Ubiquinone Oxidoreductase Subunit C2	-0.35	4.07E-02
MRPS36	Mitochondrial Ribosomal Protein S36	-0.46	4.19E-02
HMGCL	3-Hydroxy-3-Methylglutaryl-CoA Lyase	-0.55	4.36E-02
SLC25A6	Solute Carrier Family 25 Member 6	0.67	4.49E-02
ALDH1L1	Aldehyde Dehydrogenase 1 Family Member L1	-0.88	4.64E-02
SLC25A42	Solute Carrier Family 25 Member 42	-0.70	4.70E-02
ECI1	Enoyl-CoA Delta Isomerase 1	-0.65	4.72E-02
STARD7	StAR Related Lipid Transfer Domain Containing 7	-0.55	4.81E-02
NT5M	5'.3'-Nucleotidase. Mitochondrial	-0.51	4.87E-02

Table S3. Metabolic Mitopathways with the number of differentially expressed genes (DEGs) involved (from inclusion body myositis (IBM) muscle RNA seq).

	**nº DEGs / nº total genes per pathway**	**Downregulated genes in the pathway**	**Upregulated genes in the pathway**	**% DEGs / total genes per pathway**	**Interactions (nº DEGs in >1 pathway/nº DEGs per pathway)**
Carbohydrate metabolism	42/74	38	4	56.8	17/42
Metals and cofactors	65/123	57	8	52.8	20/65
Vitamin metabolism	22/49	17	5	44.9	14/22
Electron carriers	7/16	7	0	43.8	7/7
Amino acid metabolism	37/90	33	4	41.1	19/37
Lipid metabolism	48/123	39	9	39.0	19/48
Nucleotide metabolism	15/41	11	4	36.6	4/15
Sulfur metabolism	4/11	3	1	36.4	2/4
Detoxification	15/51	10	5	29.4	5/15

Table S4. Metaboanalyst analysis combining metabolic differentially expressed genes (DEGs) in muscle RNA-seq with organic acids and nucleotides in urine. Significant pathways from pathway analysis and network enrichment analysis represented in the table.

**Pathway analysis DEGs + organic acids**	**Total genes in pathway**	**Hits matched**	**FDR**
Citrate cycle (TCA cycle)	42	24	1.96E-19
Valine. leucine and isoleucine degradation	88	23	7.73E-10
Propanoate metabolism	48	17	1.39E-09
Pyruvate metabolism	45	12	3.01E-05
Butanoate metabolism	29	9	1.37E-04
Glyoxylate and dicarboxylate metabolism	56	11	1.29E-03
Synthesis and degradation of ketone bodies	10	4	1.34E-02
Alanine. aspartate and glutamate metabolism	61	9	3.64E-02
Nicotinate and nicotinamide metabolism	42	7	4.49E-02
Fatty acid degradation	102	12	4.49E-02
**Network enrichment analysis pathways DEGs + organic acids**			
Citrate cycle (TCA cycle)	78	6	3.63E-06
Alanine. aspartate and glutamate metabolism	98	4	7.90E-03
**Pathways analysis DEGs+ nucleotides**			
Citrate cycle (TCA cycle)	42	20	2.04E-13
Propanoate metabolism	48	16	5.31E-08
Valine. leucine and isoleucine degradation	88	21	7.87E-08
Pyruvate metabolism	45	10	2.15E-03
Glyoxylate and dicarboxylate metabolism	56	10	9.72E-03
Butanoate metabolism	29	7	9.72E-03
Synthesis and degradation of ketone bodies	10	4	1.65E-02
**Network enrichment analysis pathways DEGs + nucleotides**			
Purine metabolism	274	11	1.86E-07
Pyrimidine metabolism	139	7	4.90E-05
